# A pilot study comparing in-person and web-based motivational interviewing among adults with a first-time DUI offense

**DOI:** 10.1186/s13722-015-0039-0

**Published:** 2015-09-03

**Authors:** Karen Chan Osilla, Susan M. Paddock, Thomas J. Leininger, Elizabeth J. D’Amico, Brett A. Ewing, Katherine E. Watkins

**Affiliations:** RAND Corporation, 1776 Main Street, Santa Monica, CA 90407-2138 USA; Department of Statistical Science, Duke University, Box 90251, Durham, NC 27708-0251 USA

**Keywords:** DUI, Motivational interviewing, Brief intervention, Computer and web intervention, Alcohol dependence

## Abstract

**Background:**

Driving under the influence (DUI) is a significant problem, and there is a pressing need to develop interventions that reduce future risk.

**Methods:**

We pilot-tested the acceptance and efficacy of web-motivational interviewing (MI) and in-person MI interventions among a diverse sample of individuals with a first-time DUI offense. Participants (N = 159) were 65 percent male, 40 percent Hispanic, and an average age of 30 (SD = 9.8). They were enrolled at one of three participating 3-month DUI programs in Los Angeles County and randomized to usual care (UC)-only (36-h program), in-person MI plus UC, or a web-based intervention using MI (web-MI) plus UC. Participants were assessed at intake and program completion. We examined intervention acceptance and preliminary efficacy of the interventions on alcohol consumption, DUI, and alcohol-related consequences.

**Results:**

Web-MI and in-person MI participants rated the quality of and satisfaction with their sessions significantly higher than participants in the UC-only condition. However, there were no significant group differences between the MI conditions and the UC-only condition in alcohol consumption, DUI, and alcohol-related consequences. Further, 67 percent of our sample met criteria for alcohol dependence, and the majority of participants in all three study conditions continued to report alcohol-related consequences at follow-up.

**Conclusions:**

Participants receiving MI plus UC and UC-only had similar improvements, and a large proportion had symptoms of alcohol dependence. Receiving a DUI and having to deal with the numerous consequences related to this type of event may be significant enough to reduce short-term behaviors, but future research should explore whether more intensive interventions are needed to sustain long-term changes.

## Background

Driving under the influence (DUI) is a significant problem. Injury from alcohol-related motor vehicle crashes is a leading cause of premature death and disability [1]. Even after individuals with a first offense attend required alcohol education programs, rates of recidivism are high [2, 3]. Despite a decline in recidivism between 1990 and 1996 in California, rates of DUI incidents in the state have remained stable since 2010.

In California, individuals with a first-time DUI conviction must complete a state-licensed DUI program in order to regain their driver’s license [4]. Programs are didactic and lecture-based and provide strategies to reduce drinking and driving, education about alcohol, and presentations by panels of victims whose lives have been affected by a DUI incident [5]. Unfortunately, DUI programs have had only modest effects on recidivism [4]. In 2011, there was no significant difference in the rates of 1-year crash and DUI incidents in California between individuals with a DUI conviction who were court-assigned to a DUI program and those who were not [6]. This finding is consistent with the general literature showing that educational-type lectures do not have any effect on behavior change among individuals with alcohol use disorders [7–9].

A more effective approach towards behavior change may be a counseling method that focuses on exploring an individual’s reasons for change and helping them develop a change plan that is meaningful for them. Motivational interviewing (MI) is a collaborative counseling style that strives to strengthen a client’s commitment to change [10]; it is grounded in theories of self-determination [11] and self-efficacy [12]. Treatment approaches grounded in these theories empower an individual’s motivation to change, reaffirm their autonomy, and guide people toward change if they are ready. MI could be particularly acceptable among individuals with DUI convictions who may vary considerably in their motivation to change. For example, some individuals may be ambivalent about changing their drinking and driving behaviors, whereas others may be very motivated to change because of the adverse event or “teachable moment” they experienced [13, 14].

MI is flexible and tailors the intervention content based on the individual’s readiness to change. Counselors use processes such as engaging (establishing a connection), focusing (establishing goals such as behavior change), evoking (eliciting the client’s motivations to change), and planning (committing to change and developing a plan) to individualize sessions [10]. Within the context of a DUI program, individuals who are not ready to change may benefit most from counselors who spend time engaging the individual and establishing rapport; individuals in later stages or those who are already moving toward change may be better helped by planning when to use problem-solving strategies and in ways to increase their commitment to action [10, 15].

Although many studies have shown the effectiveness of MI in other settings (e.g., college, primary care, substance abuse treatment) compared to no treatment [16], in-person interventions are limited by the availability of trained counselors, training costs, and the challenge of implementing the approach uniformly [17]. For example, research shows that counselors attending MI workshops often lose MI proficiency over time unless they receive ongoing feedback and coaching post-training [18, 19]. Thus, it is important to think about ways to utilize MI that may be more cost-effective and consistent. Web-based interventions that utilize MI principles (web-MI) may be a promising approach.

Web-MI makes it possible to disseminate evidenced-based approaches with high fidelity because the content is programmed and automated. Web-MI has been shown to be effective in reducing at-risk drinking among college students [20, 21] and adults in the general population and military [22, 23]. Web-MI has also been effective in reducing smoking among English and Spanish speakers [24], as well as drug use among postpartum women [25]. While some web-MIs are being explored in criminal justice settings [26], web-MI has not been tested in DUI settings. Few studies have compared the effectiveness of web-MI to active comparison groups (i.e., other interventions that include alcohol content) [27]. Existing research studies have typically compared the effects of a stand-alone web-MI to an assessment-only group [28], and they generally show small effect sizes at short-term follow-up [29]. Although even small effects may be clinically meaningful, more research is needed to improve their comparative effectiveness and determine whether these interventions may serve as an adjunct to more intensive approaches.

The current study evaluated the acceptance and efficacy of web-MI and in-person MI interventions among a diverse sample of individuals with a first-time DUI offense. This Stage 1b trial focused on determining participant acceptance of the intervention and intervention feasibility, and predicting the likely size of intervention effects for future trials [30]. We randomly assigned individuals enrolled in a DUI program to usual-care (UC-only), in-person MI plus UC, or web-MI plus UC. Given the pilot nature of this work, our primary aims were to evaluate the acceptance and efficacy of these MI interventions on alcohol-related outcomes compared to UC-only. We hypothesized that participants in the in-person and web-MI interventions would have greater acceptance and reduced alcohol consumption and alcohol-related consequences compared to UC-only.

## Methods/design

### Setting and design

Project REACH (Rethinking Avenues for Change; in Spanish, *REtomondo Avenidas para el Cambio Hoy*) was conducted in collaboration with the Los Angeles County Alcohol and Drug Program Administration (ADPA) and three private DUI programs under ADPA’s regulatory authority. All clients received UC. Consenting clients were randomized to one of three conditions: UC-only, UC plus in-person MI, or UC plus web-MI using randomized block sampling with equally-sized blocks of six.

All procedures were approved by the institution’s IRB. Because of the sensitivity of collecting data while clients were enrolled in the DUI program, participants were told that our Certificate of Confidentiality protected their privacy from any civil, criminal, administrative, legislative, or other proceeding at the federal, state, or local level and that participation in the study would not affect the services to which they were entitled.

### Study conditions

#### Usual care

UC consisted of nine 2-h group sessions, twelve 1-h educational classes, and six community-based 12-step meetings. The 2-h group sessions were unstructured support groups that encouraged participants to examine their own personal attitudes and behavior and receive support for their alcohol or drug problems [31]. In the beginning of the study, we conducted focus groups with UC clients who reported that sessions were focused mainly on the consequences of DUI and heavy drinking [32].

#### Intervention conditions

We conducted focus groups with DUI program staff and clients to develop the in-person MI intervention for this population. Next, we adapted the in-person intervention for the web, and then conducted individual usability testing interviews with web-MI clients who were already enrolled in the DUI program [32]. We used the usability feedback to iteratively revise the interventions. We developed the interventions simultaneously in English and Spanish in order to create interventions that were culturally equivalent. The current pilot study evaluates the final revised intervention created from these formative assessment procedures.

Both the in-person MI and web-MI interventions consisted of one 45-min individual session and two 10-min booster sessions that were delivered by the same facilitator. Each session was different. The goals of the MI interventions were to reduce drinking and alcohol-related problems. The content of both the in-person and the web-MI intervention was adapted from earlier MI work [33–35] and covered similar content, but the efficacy of the revised interventions had not been tested until the current pilot study. The first session included normative personalized feedback in the following areas: (1) how their drinking and estimates of others’ drinking compared to other men/women their age [36]; (2) their positive beliefs about drinking and the balanced placebo design experiment, which describes how alcohol expectancies (i.e., actual vs. expected effects) can influence drinking [37]; (3) their negative consequences from drinking, including their estimated blood alcohol content values; and (4) strategies for avoiding consequences in the future. Participants who were ready to change their drinking were asked to discuss a drinking-related goal they wanted to work on before the next booster session. We then used rulers to assess their confidence and willingness to work on that related goal. If the participant was not ready to change, the facilitator went straight to the rulers.

The in-person and web-MI booster sessions were formatted similarly and included a check-in about the participant’s drinking and goals from the previous session (e.g., “Last session, you said that you would try to stop your drinking by not going to happy hour. How did that go for you?”). Booster sesssions provided an opportunity to talk about new strategies to stop drinking, if they were willing, and to discuss their confidence and willingness to change using rulers with a 1–10 scale (e.g., not confident to very confident).

The style of the MI interventions was as important as the content. Both web-MI and in-person MI interventions used core MI skills, such as open-ended questions, affirmations, reflective statements, and summaries to convey a nonjudgmental and non-confrontational style [10]. For example, the in-person MI manual had examples of open-ended questions and reflective statements that facilitators could use, and these same statements were used by the narrator in the web-MI intervention. Both interventions emphasized the underlying spirit of MI (e.g., collaboration, evocation, acceptance, compassion).

While the web-MI intervention incorporated the same sections of the in-person MI intervention, we used artificial intelligence (e.g., automatic responses tailored to the participant’s responses) and personalized feedback to tailor the intervention to the participant so that each session was interactive. For example, we used audio recordings and videos to share personalized feedback from the participant’s baseline survey (e.g., “We asked you what your drinking was like and you said you drank 4 days a week.”); asked questions that participants could respond to and receive tailored audios/videos based on those responses [e.g., “What do you think of this information?” (participant clicks “I’m surprised”); “It is very common to be surprised by this information and wonder if the numbers are correct…”]; used interactive exercises (“Click a number that best describes your mood when you start to drink. What happens to your mood as you continue to drink?”); and elicited change talk (e.g., “For the confidence ruler, why a 4 and not a 0?”). The web-MI intervention was narrated by a female Latina, and text captions were also available at the bottom of the screen. It was programmed at a 5th-grade reading level.

### Participants and recruitment

**Fig. 1:**
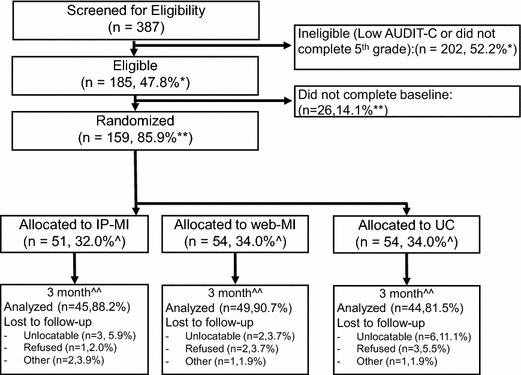
CONSORT diagram. *Denominator is total screened for eligibility (n = 387); **denominator is number eligible (n = 185); ^denominator is number randomized (n = 159); ^^denominator is number allocated to each group (IP-MI n = 51, web-MI n = 54, UC n = 54)

Participants were individuals 21 and older convicted of a first-time DUI offense and who had entered one of the three participating 3-month DUI programs. Upon enrolling in the program, program staff asked clients if they could be contacted about a research study. Interested clients completed a consent-to-contact form. Consenting clients were contacted and screened for 5th-grade completion (because of the nature of our self-report instruments) and at-risk drinking, using a score of 3 or higher for women or 5 or higher for men on the Alcohol Use Disorders Identification Test consumption questions (AUDIT-C; [38]). These cut points perform well, with high sensitivity and specificity in screening for at-risk drinking in the general population (men: >90 %, women: 80 %; [39]). Approximately 52 percent were ineligible for the study based on low AUDIT-C score and education (see Fig. 1).

Participants (N = 159) were randomized to one of the three conditions. Participants assigned to either of the two MI interventions were asked to complete their first individual session by the third week of entering the DUI program, and their booster sessions by their ninth and eleventh week in the DUI program, respectively. The timing of these intervention sessions was meant to correspond with the beginning, middle, and end of a participant’s 3-month DUI program. As noted earlier, participants in both MI interventions had the same number of sessions as participants in the UC-only condition because their MI sessions replaced two 12-step meetings. All MI intervention sessions were delivered in a private office at the DUI program where UC groups took place. A total of 138 participants were successfully assessed at follow-up (86.8 %).

### Procedures

#### Client data collection

We used in-person interviews to assess DSM-IV criteria for past-year alcohol abuse and dependence. Participants also completed web-based surveys at baseline, after the first session, and 3 months after baseline or at program termination. Participants received a $25 gift card for the Alcohol Use Disorder and Associated Disabilities Interview schedule (AUDADIS; [40]), $25 for the baseline survey, $10 for a satisfaction survey immediately after their first session, and $50 for the 3-month follow-up. All clients were followed in our intent-to-treat analyses, regardless of whether they completed all intervention sessions.

#### Facilitator training and supervision

Three bilingual facilitators received 40 h of MI training that included a one-day MI workshop delivered by authors KCO and EJD, who are clinical psychologists affiliated with the Motivational Interviewing Network of Trainers. In addition, facilitators received additional coaching and feedback after each session from KCO, who listened to audio recordings of sessions.

#### Intervention fidelity data collection and coding

In-person MI sessions were audio recorded. Two independent coders received 40 h of coding training [41], which included a half-day training on the Motivational Interviewing Treatment Integrity (MITI) scale [42] and several MITI practice assignments with pre-coded audio recordings [43]. Raters met weekly to discuss coding discrepancies and reconcile questions to maintain inter-rater agreement. UC sessions were not coded because not all individuals attending these groups were enrolled in the study, so it was not possible to record these sessions.

To monitor web-MI intervention fidelity, our web program measured the number of minutes each participant spent in each session. A facilitator was present in the room to address any problems that might have emerged, which may have also helped to ensure that the participant completed the session.

### Measures

At baseline, we collected demographics and alcohol abuse and dependence information using AUDADIS and AUDIT-C [38, 40]. We collected client acceptance/satisfaction data immediately following the first session. At baseline and at 3-month follow-up, we collected data on alcohol use.

#### Client ratings of quality and satisfaction

Participants in all three conditions answered questions about the quality of and their satisfaction with the experience. Participants were asked, “How would you rate the quality of your session?” on a 4-point Likert scale, with a higher score representing higher quality. Satisfaction was measured using 22 items that were averaged (e.g., This program was respectful of my background; I felt the program respected where I was at with my alcohol and that any change was up to me; The program valued my opinion). Additional items included questions about the usefulness, quality, impact, and helpfulness of the session. They also were asked nine questions rating their session facilitator [44].

#### MI intervention fidelity

MITI 3.1 was used to code competency and adherence to in-person MI, and integrity was measured through global scores and behavioral counts [42]. The MITI 3.1 has five global scales (evocation, collaboration, autonomy/support, direction, and empathy) that are scored from 1 (low) to 5 (high), with a score of 3.5 indicating beginning proficiency and 4 indicating competency. The rater also counts the number of specific behaviors that occur during each coded segment, including the number of open questions and closed-ended questions, MI-adherent and nonadherent statements, and simple and complex reflections. Whereas global scores have a limited range (1–5), behavioral counts utilize a running tally with no upper end on the scale; thus, these scores can vary by session.

Twenty percent of the in-person MI sessions (n = 10) were randomly selected for double-coding. We calculated prevalence-adjusted, bias-adjusted kappa (PABAK; [45] to assess inter-rater agreement for each global score by dichotomizing the 1–5 scale into 1–3 (MI beginning proficiency) and 4–5 (MI competent). The PABAK scores for the global scores of evocation, collaboration, autonomy/support, direction, and empathy were 0.6, 0.2, 0.6, 1.0, and 0, respectively, while the agreement averaged 72 percent across the global scores. We also calculated intraclass correlations (ICCs) between raters for each behavioral count. These ICCs ranged from 0.30 (MI-adherent) to 0.91 (closed questions), and averaged 0.69 across the behavioral counts. Since the distribution of MI-nonadherent behavioral counts was skewed toward 0 (with only one value among the coders that was neither 0 nor 1), a kappa statistic was computed instead to assess inter-rater agreement for reporting any versus no MI-nonadherent behaviors (PABAK = 0.2).

#### Client outcomes

Outcomes included changes in drinking behaviors and related consequences in the past 3 months. We examined the intensity and frequency of drinking in the past 3 months [46]. Drinking frequency was measured by asking how often participants drank alcohol in the past 3 months. Reponses ranged from 0 (‘Never’) to 10 (‘Every day’). We converted these response categories to a pseudo-continuous variable to easily interpret the results as the number of days. Drinking frequency ranged from 0 to 90 days (e.g., ‘Never’ = 0 days, ‘Less than Once a Month’ = 2 days). Drinking quantity was measured by asking the respondent the typical number of drinks on a given occasion. Days of reported heavy drinking, defined as four or more drinks for women and five or more drinks for men, was also transformed from a categorical variable to a pseudo-continuous variable ranging from 0 to 90 days, as described above for drinking frequency. Drinking and driving in the past 3 months was reported on a categorical scale ranging from 0 (‘Never’) to 10 (‘Every day’). Due to the skewed distribution of this variable, we created a dichotomous version to indicate any drinking while driving in the past 3 months. We assessed negative consequences from alcohol use using the Shortened Inventory of Problems Modified for Alcohol and Drug Use [47]. Finally, marijuana use frequency was assessed by asking participants how often they used marijuana in the past 3 months. Reponses were transformed to a continuous variable ranging from 0 to 90 days.

### Analytic strategy

#### Client ratings of session quality and satisfaction

Client quality and satisfaction data were analyzed for differences across the three conditions using ANOVA and pair-wise *t*-tests.

#### Preliminary intervention efficacy

We first examined whether there was significant change over time in the outcomes within each study condition by conducting Wilcoxon signed-rank tests on the difference scores of continuous outcomes. We conducted a McNemar’s test to assess a significant change in the rate of obtaining a DUI and experiencing negative consequences (none vs. any) in the past 3 months between baseline and follow-up, within study condition. Pseudo-continuous variables were treated as continuous, given the assumption that each variable reflected an underlying continuum [48, 49]. The treatment of these variables as continuous were expected to result in low bias when measuring more than seven categories and the measure had a bell-shape [50]. When the latter condition was not met, we ran sensitivity analyses with the ordinal outcome to confirm that conclusions did not differ under the two model specifications.

We next conducted analyses to test for a significant intervention effect. All outcomes were analyzed using an intent-to-treat approach. To compare the baseline characteristics of clients assigned to each condition, we used Chi squared tests for categorical variables and one-way ANOVAs for continuous variables. Longitudinally, each outcome was modeled with generalized, linear, mixed-effects regression modeling using the GLIMMIX procedure in SAS software (Version 9.2). Covariates included in the model were those characteristics identified as significant (p < 0.1) in bivariate analyses with the outcomes. These included days of marijuana use in the last 3 months and average number of drinks [46]. The baseline value of the outcome was included as a covariate in all models to control for any important differences among conditions and to improve the precision of the intervention-effect estimates [51]. Dummy variables for web-MI and in-person MI, with the comparison condition as the hold-out category, were included in all models. When the distribution of the pseudo-continuous measures was not bell-shaped, ordinal logistic regressions were conducted on the original ordinal measure as a sensitivity analysis to confirm that analytic conclusions did not differ under the two model specifications.

## Results

### Client acceptance

Approximately 57 percent attended all three in-person MI sessions, 14 percent attended two sessions, and 29 percent attended one session. Approximately 65 percent attended all three web-MI sessions, 17 percent attended two sessions, and 18 percent attended one session. We did not have data on the number of UC sessions attended. Participants’ ratings of session quality varied significantly across conditions [*F*(2135) = 6.93, *p* = 0.0014]. On average, in-person MI participants rated the quality of their session highest compared to web-MI and UC-only, and there were no differences in ratings by facilitator. Quality ratings were next highest for participants in the web-MI, and then the UC-only condition. Participants in the in-person MI intervention rated their satisfaction with the sessions significantly higher than participants from the other two conditions. There were no significant differences in satisfaction between web-MI and UC participants.

### Intervention fidelity

Facilitators of the in-person MI scored a mean of 4.2 on the MITI global scores (*SD* = 0.1; range: 3.9–4.5), which indicates MI competency. Behavioral counts ranged between 5.5 (giving information), 9.5 (simple reflections), and 11.1 (MI-adherent statements, complex reflections), suggesting high frequency of MI-consistent behaviors.

### Intervention efficacy

#### Sample characteristics

Sixty-five percent of the participants were male, 40 percent were Hispanic/Latino/a, 87 percent were born in the United States (excluding Puerto Rico), 91 percent had at least a high school education, and 64 percent were fully employed. Participants were 30.0 (SD = 9.8) years of age (Table 1).

**Table 1:** Baseline characteristics of the study sample

	UC (N = 54)		In-person MI (N = 51)		Web-MI (N = 54)	
	Mean (%)	SD	Mean (%)	SD	Mean (%)	SD
Male	64.81		68.63		62.96	
Race						
Hispanic/Latino	38.89		41.18		40.74	
African American	9.26		9.80		11.11	
White	35.19		39.22		31.48	
Asian/PI	7.41		5.88		11.11	
Place of birth						
US, except Puerto Rico	85.19		92.16		85.19	
Other	14.81		7.84		14.81	
Education						
<HS/GED	3.70		5.88		3.70	
HS/GED	3.70		3.92		5.56	
>HS	92.59		90.20		90.74	
Employment						
Full/part time	66.67		64.71		59.26	
Unemployed	9.26		9.80		20.37	
Other work situation	24.07		25.49		20.37	
Age at time of DUI	29.56	8.96	29.75	10.18	30.56	10.40
Alcohol use measures						
Negative consequences (SIP)	8.70	8.26	10.06	11.08	10.76	10.14
Past 12 Months						
AUDADIS alcohol dependence^	64.15		64.71		74.07	
AUDADIS alcohol abuse^	88.68		96.08		90.74	
Past 3 months						
Alcohol use # of days	25.03	24.73	31.18	28.56	25.99	23.53
# Drinks on typical occasion	4.35	2.30	4.37	1.91	5.02	3.27
Heavy drinking # of days	10.19	12.87	13.93	18.39	10.55	13.09
Drink and drive past 3 months	51.85		49.02		48.15	
# Drinks on heaviest occasion*	7.98	4.03	8.98	3.99	10.56	24.07
Marijuana use	42.59		50.98		44.44	
Marijuana use in days*	16.11	31.22	27.74	28.77	10.56	24.07
Any other drug use	3.70		9.80		12.96	

* p < 0.05; ^ 1 UC participant did not complete the AUDADIS

Overall, 92 percent of the sample met diagnostic criteria for past-year alcohol abuse. Sixty-seven percent met diagnostic criteria for dependence. Diagnoses of alcohol abuse and dependence were not significantly different across the three conditions (for abuse, Χ^*2*^(2) = 1.52, *p* = 0.468; for dependence, Χ^2^(2) = 2.00, *p* = 0.368).

At baseline, web-MI clients reported more drinks on the occasion they drank the most compared to UC-only or in-person MI clients [see Table 1; *F*(2156) = 3.39, *p* = 0.036]. Clients receiving in-person MI also reported more days of marijuana use in the past 3 months compared to UC-only and web-MI clients [*F*(2156) = 3.97, *p* = 0.021]. Only one participant in our sample was monolingual Spanish-speaking, and was assigned to the web-MI intervention and included in analyses. There were no significant differences in outcomes by ethnicity.

#### Alcohol-related outcomes

Table 2 shows differences in within-group outcomes between baseline and follow-up. Overall, participants from all three conditions reported reduced drinking quantity, alcohol-related consequences, and drinking and driving between baseline and 3-month follow-up (p < 0.05). Regarding drinking outcomes, all participants drank about one drink less on a typical occasion compared to baseline amounts (p < 0.05) and reported fewer and less frequent consequences (3- to 4-point reduction roughly translates to experiencing several problems weekly to experiencing problems a few times in the past 3 months). Web-MI participants reported drinking 4.58 fewer days in the past 3 months at follow-up compared to baseline (p = 0.036). UC-only and in-person MI clients did not report a significant decrease in drinking days.

**Table 2:** Differences in within-group outcomes between baseline and follow-up

Variable	UC				In-person MI				Web-MI			
	Difference (SD)	Test stat*	*d* ^+^	*p*	Difference (SD)	Test stat*	*d* ^+^	*p*	Difference (SD)	Test stat*	*d* ^+^	*p*
Negative consequences (SIP)	3.82 (8.81)	186.50	0.46	0.013	3.24 (6.10)	233.00	0.29	0.001	3.20 (5.86)	326.50	0.32	<0.0001
Alcohol use # of days	2.63 (19.12)	35.50	0.11	0.474	3.54 (17.49)	66.50	0.12	0.132	4.58 (14.30	126.50	0.19	0.036
# Drinks on typical occasion	0.61 (1.97)	111.50	0.27	0.049	0.82 (2.10)	115.50	0.43	0.014	1.08 (3.33)	143.00	0.33	0.011
Heavy drinking # of days	2.03 (15.46)	40.00	0.16	0.442	1.70 (10.90)	69.50	0.09	0.218	0.61 (13.21)	24.00	0.05	0.742

*  Wilcoxon signed rank test for continuous variables

^+^Effect size is computed as Cohen’s *d*: difference/within-group baseline standard deviation

While there were within-group reductions in alcohol-related consequences, participants in all three groups continued to report alcohol-related consequences at follow-up. The proportions of participants who reported having at least one consequence at baseline and subsequently reported at least one consequence at follow-up were 61 percent of in-person MI, 78 percent of UC-only, and 81 percent of web-MI individuals.

Participants also reported within-group changes in their drinking and driving in the past 3 months (p < 0.0001). Across the three groups, 40–56 percent of participants reported not drinking and driving at follow-up. We were specifically interested in seeing the proportion of participants who reported drinking and driving behavior at baseline and whether they continued this behavior at follow-up. Across all conditions, about 50 percent of participants reported drinking and driving in the 3 months prior to baseline.^1^ Of those participants, about 8 percent of them reported drinking and driving at follow-up. Also important to note, of participants who reported no drinking and driving within 3 months of baseline, 96 percent of them continued to report no drinking and driving at follow-up.

Table 3 shows the estimated intervention effect of each MI + UC condition compared to UC-only. After adjusting for baseline levels, there were no significant group differences between the MI conditions and the UC-only condition in alcohol consumption (number of typical and heavy drinking days, average number of drinks) and risk behaviors (alcohol-related negative consequences and drinking and driving). Further, estimates of the effect sizes were small (Cohen’s *d* = 0–0.12) for typical and heavy drinking days, average number of drinks, and alcohol-related consequences. For drinking and driving in the past 3 months, the width of the confidence intervals indicates that substantial variability exists in our estimates.

**Table 3:** Intervention effect estimates of outcomes compared to usual care at 3 months post-baseline

Outcome	Estimate	Confidence interval		t statistic (132 df)	*p* *value*	d*
Negative consequences (SIP)						
Web-MI	1.13	−1.42	3.69	0.88	0.382	0.12
In-person MI	1.13	−1.47	3.72	0.86	0.392	0.12
Alcohol use # of days						
Web-MI	0.03	−6.15	6.20	0.01	0.993	0.00
In-person MI	0.48	−5.79	6.75	0.15	0.880	0.04
# Drinks on typical occasion						
Web-MI	0.00	−0.81	0.81	0.00	0.998	0.00
In-person MI	−0.01	−0.82	0.81	−0.01	0.988	0.00
Heavy drinking # of days						
Web-MI	1.29	−3.31	5.90	0.56	0.580	0.08
In-person MI	1.20	−3.47	5.87	0.51	0.612	0.09

	Log-odds ratio	Confidence interval		t statistic (132 df)	*p* *value*	Odds ratio
Drink and drive past 3 months						
Web-MI	−1.39	−12.78	10.00	−0.24	0.810	0.25
In-person MI	−1.99	−13.43	9.46	−0.34	0.732	0.14

* Cohen’s *d* = estimate/pooled standard deviation across the two comparison conditions

## Discussion

This pilot study takes an important first look at the acceptance and efficacy of new in-person MI and web-MI interventions added to DUI UC compared to UC-only for a diverse sample of individuals enrolled in a first-time DUI program. Participants in both the in-person and web-MI intervention conditions rated the quality of and satisfaction with their session higher than participants in the UC-only condition, suggesting that clients were more receptive to the MI interventions. Clients viewed the in-person MI more favorably than web-MI, which may be related to the stronger therapeutic alliance often found in the in-person interactions compared to web-based interactions [52]. However, we did not find statistically significant differences in outcomes between the MI conditions and UC-only condition. In fact, regardless of study condition, participants reported significant reductions in both alcohol consumption and risk behaviors. Thus, at program completion, participants from all three study conditions reported reduced alcohol consumption, DUI, and fewer alcohol-related consequences.

There were at least two unexpected results from our study. First, despite recruiting individuals with a first-time offense into the study, 67 percent of our sample met criteria for alcohol dependence, and the majority of participants in all three study conditions continued to report alcohol-related consequences at follow-up. From our previous discussions with DUI providers, we anticipated a larger percentage of at-risk versus dependent drinkers, and therefore designed the MI intervention for an at-risk population rather than a population with dependence. Our second unexpected result was that participants from each of the MI interventions did not report differences in their outcomes when compared to UC-only. We had hypothesized that because our MI interventions were focused on exploring behavioral change and developing a change plan, we would see significant improvements in individuals who received the MI interventions compared to individuals who only received UC.

There are several possible explanations for these findings. First, the extensiveness of UC services (i.e., 36 program hours) in these DUI programs may have been sufficient to improve outcomes in the short term, and an additional 3-session MI (i.e., about 65 min) may not have had an additive effect on outcomes. Second, our follow-up timeframe was short. We were only able to measure outcomes at the conclusion of clients’ 3-month DUI program. Receiving a DUI and having to deal with the numerous financial, emotional, and social consequences (e.g., vehicle impoundment, jail time, probation, injury) related to this type of event may be significant enough to reduce a client’s alcohol-related behaviors in the short term, but might not be enough to sustain long-term changes such as reductions in recidivism (D’Amico et al. [13]). This speaks to the challenge of conducting research in DUI programs that have strong behavioral expectations and high sanctions for failing those expectations. Longer follow-up assessments (e.g., 6 months to one year after program completion) may be needed to better understand whether MI interventions and UC are differentially associated with sustained behavior change after a client completes a DUI program. Finally, MI interventions may not be the best fit for individuals with a first-time DUI offense, given the high levels of dependence that were reported in this study. Future studies should evaluate whether alcohol dependence is as common in other first-time DUI offense programs. If dependence rates are similarly high in other programs, more intensive treatment approaches such as cognitive behavioral therapy or medication-assisted therapy may be more effective [53–58]. MI interventions may still be used to enhance engagement prior to these more intensive and long-term approaches [59] or they may be a better fit as a preventive intervention with individuals who are at risk for a future DUI but who have not yet been convicted. Determining which therapy or combination of therapies is associated with long-term changes should be the subject of future research.

In conducting community-based work, it is always important to examine lessons learned to help inform future research. Although MI is an evidence-based treatment that has been successful as a brief intervention in a variety of settings, it may be important to have more sessions for this more severe population. The population and providers were very receptive to MI [32], and it could perhaps be integrated into the lengthy UC treatment and provided in this group setting as with other mandated populations [13]. Given that clients felt that the MI intervention was of higher quality and were more satisfied with it than UC, integrating MI into UC could help standardize services provided to clients, help make the program more acceptable to them, and perhaps increase attendance, which could lead to better outcomes.

Our sample was recruited from DUI programs in California and may not be representative of clients in DUI programs nationally. Of note, about 52 percent of our sample was excluded mostly due to low AUDIT-C at program entry, which also affects generalizability. In addition, inter-rater agreement on the MITI was low for five measures, suggesting improvements in measure performance and/or the process for coding those items are needed for future studies. It is also important to note that under-reporting of DUI behaviors might be an issue, given the setting. The limitations of self-report data are well-known, although much research has shown that self-report is valid when procedures such as those used in the current study are implemented (e.g., establishing rapport and discussing confidentiality) [60].

This pilot study addresses an important policy question by examining whether individuals with a first-time DUI offense find MI interventions acceptable in a DUI setting and whether clients who receive an MI intervention have improved outcomes relative to UC. Findings suggest that participants from all three conditions experienced improved outcomes, regardless of study condition. Although it is possible that a longer follow-up may provide insights into whether within-group differences are sustained, we hypothesize based on our effect sizes and confidence intervals that between-group differences in a larger future trial with this population are not supported by the data. Instead, given that individuals with a first-time DUI offense are likely a unique population of individuals who may be experiencing consequences related to alcohol dependence, future research is needed to better understand the potential heterogeneity of this population and to determine the most appropriate level of care for these high-risk individuals to reduce long-term recidivism.

## Footnotes

1 In some cases, individuals might enter the DUI program more than 3 months after their DUI arrest.
